# The Association Between White Blood Cell Count and Insulin Resistance in Community-Dwelling Middle-Aged and Older Populations in Taiwan: A Community-Based Cross-Sectional Study

**DOI:** 10.3389/fmed.2022.813222

**Published:** 2022-02-17

**Authors:** Jau-Yuan Chen, Yun-Hung Chen, Yu-Chien Lee, Meng-Ting Tsou

**Affiliations:** ^1^Department of Family Medicine, Chang-Gung Memorial Hospital, Taoyuan City, Taiwan; ^2^College of Medicine, Chang Gung University, Taoyuan, Taiwan; ^3^Department of Epidemiology, Harvard T.H. Chan School of Public Health, Boston, MA, United States; ^4^Department of Family Medicine, Mackay Memorial Hospital, Taipei City, Taiwan; ^5^Department of Occupation Medicine, Mackay Memorial Hospital, Taipei City, Taiwan; ^6^Department of MacKay Junior College of Medicine, Nursing, and Management, New Taipei City, Taiwan

**Keywords:** insulin resistance, anthropometry, cardio-metabolic risk factors, prediction, white blood cell count

## Abstract

**Background:**

Insulin resistance (IR) is a major pathophysiological factor in the development and progression of diabetes mellitus (DM). DM is highly prevalent in Taiwan and has become one of the most common health problems in family medicine and primary care. We aimed to use white blood cell count (WBC), a common physiological parameter, to develop a simple clinical prediction rule for IR in the middle-aged and old Taiwanese population.

**Methods:**

In this cross-sectional community-based study, the participants completed a questionnaire comprising personal and medical history data and underwent anthropometric measurements and blood sampling. IR was defined as a HOMA-IR index ≥2. Independent *t*-test, Mann–Whitney U test, chi-square test, Pearson's correlation test, multivariate binary logistic regression, and receiver operating characteristic curves were used to evaluate the association between the WBC count and IR.

**Results:**

A total of 398 community-dwelling middle-aged and older persons (34.9% men) with a mean age of 64.43 ± 8.45 years were enrolled for the analysis. A significant association was identified between the WBC counts and IR, with a Pearson's correlation coefficient of 0.37 (*p*-value <0.001). Multivariate logistic regression revealed that WBC count (OR = 1.50; 95% CI = 1.25–1.81) was an independent risk factor for IR after adjusting for confounding variables. The area under the receiver operating characteristic curve for WBC count was 0.67, and the optimal threshold value was 5.65 1,000/uL.

**Conclusion:**

A high WBC count is positively related to an increased risk of IR among middle-aged and older people in Taiwan.

## Introduction

Insulin resistance (IR) is recognized as a serious public health problem worldwide and has emerged as a major pathophysiological factor in the development and progression of type 2 diabetes mellitus (T2DM) and metabolic disease (1–4). The hyperinsulinemic euglycemic clamp test, the gold standard for assessing IR, has been used to quantify IR using a variety of methods. However, this test is expensive, invasive, and time-consuming, making it unsuitable for clinical purposes or large-scale studies (5). Therefore, the homeostasis model assessment of IR (HOMA-IR) is used to evaluate IR (6, 7).

According to a national study, the prevalence of T2DM between 2017 and 2020 was 11.05% in Taiwan (8). As IR is a risk factor for the development of T2DM, the early identification of IR is important from a public health perspective. Currently, there are few standard methods for quantifying IR. The lack of standardized insulin assays has limited the clinical utility of HOMA-IR, although it has been widely used in the study of metabolic syndrome (9). Although HOMA-IR is used for quantifying IR, it is not routinely conducted in clinical practice. Therefore, a simple and more accessible marker for predicting IR may be useful for the early identification of individuals with IR. In clinical practice, evaluating IR using the HOMA-IR index is often difficult as insulin and fasting glucose levels are not routinely measured.

Growing evidence indicates that IR is closely associated with chronic subclinical inflammation (10–12). Biomarkers associated with inflammation, such as white blood cell (WBC), C-reactive protein and interleukin-6 levels, are considered as independent predictors of cardiovascular disease and T2DM in adults, (13–15). Among these biomarkers, the WBC count is commonly measured and is more cost-effective. However, few studies have examined the association between WBC count and IR measured by HOMA-IR. Some studies have proposed that the circulating WBC count is a biomarker for the prognoses of cardiovascular risk and IR (16–18), but the possibility of identifying IR using elevated WBC counts has not been fully investigated.

The major purpose of this study was to establish a simple clinical marker for the early identification of IR in the middle-aged and older populations in Taiwan. Additionally, our study results may provide valuable information for primary care physicians to alert subjects in this age group regarding the increased risk of IR.

## Materials and Methods

### Study Design and Participants

This was a cross-sectional, community-based study conducted between January and October 2014. Data for this study were collected from a community health promotion project of the Linkou Chang Gung Memorial Hospital. We recruited 619 volunteers aged ≥50 years from eight cluster-randomized of 28 villages in Guishan district, Taoyuan city. The inclusion criteria were as follows: (1) subjects aged 50 years or above and (2) subjects residing in Guishan district for at least half a year. The exclusion criteria were as follows: (1) subjects with functional disability, (2) subjects who declined to participate, (3) subjects who could not complete all the examinations or face-to-face interviews, and (4) subjects with outliers of HOMA-IR level. A total of 398 participants, including 139 men and 259 women, were included in the analysis. Each participant completed a questionnaire that included personal information and medical history through face-to-face interviews conducted by trained research assistants. The project was approved by the Institutional Review Board of Linkou Chang Gung Memorial Hospital (102-2304B), and all the participants were fully informed and signed informed consent forms before enrollment.

### Anthropometric and Laboratory Examinations

After obtaining informed consent, detailed anthropometric measurements (such as height, weight, age, and waist circumference [WC], and blood sampling were conducted by trained research nurses under the supervision of a medical doctor. Height was measured using calibrated height meters while the participant stood erect and barefooted, with feet placed together and facing forward. Body mass index (BMI) was calculated as weight divided by the square of the height (kg/m^2^). The WC was measured at the level midway between the iliac crest and the lower border of the 12th rib while the participant stood with feet 25–30 cm apart. Blood pressure was measured using an automated sphygmomanometer after a 10-mins rest in a seated position, and the lowest reading was recorded. WBC count and clinical biochemistry tests were performed in a hospital laboratory accredited by the College of American Pathologists; and, these included fasting plasma glucose (FPG), high-density lipoprotein cholesterol (HDL-C), low-density lipoprotein cholesterol (LDL-C), total cholesterol, triglyceride (TG), and serum creatinine levels. Venous blood samples were collected after overnight fasting for at least 12 h. A standardized biochemistry analyzer (Hitachi LST008, Hitachinakashi, Japan) was used for the determination of laboratory tests. A standardized Sysmex XN series analyzer (Sysmex XN 9000) was used for the determination of WBC.

### Definitions of Hypertension, Diabetes Mellitus, and Hyperlipidemia

Hypertension (HTN) was defined by a systolic blood pressure (SBP) ≥ 140 mmHg or a diastolic blood pressure (DBP) ≥ 90 mmHg, the current use of antihypertensive medications, or a history of HTN. Diabetes mellitus (DM) was defined as a FPG level ≥ 126 mg/dL, the use of oral antidiabeticic drugs or insulin therapy, or a history of DM. Hyperlipidemia was defined as a LDL-C level ≥ 130 mg/dL, a TG level ≥ 150 mg/dL, a total cholesterol level ≥ 200 mg/dL, the use of lipid-lowering medication, or a history of hyperlipidemia (8).

### Definition of Insulin Resistance

HOMA-IR was expressed as fasting glucose (mmol/L) × fasting insulin (mU/mL)/22.5 (19). To define the main outcome variable of IR, the HOMA-IR cut-off value was set at 2.0.

### Statistical Analysis

The sample size was determined using the G^*^power 3.1 software. The sample of this study comprised 398 subjects, which implied sufficient statistical power. All data on demographics and clinical characteristics were expressed as the mean ± standard deviation (SD) or median (Q1, Q3) for continuous variables and numbers (%) for categorical variables. Descriptive statistics were presented, and differences between the IR and non-IR groups were compared using the independent *t*-test and Mann–Whitney U test for continuous data and the chi-square test for categorical data. The correlations between the HOMA-IR index and cardiometabolic risk factors were assessed using Pearson's correlation test. In the multivariate analysis, binary logistic regression was used to adjust for covariates. Receiver operating characteristic (ROC) curves were plotted for WBC and IR. The area under the ROC curve (AUC) and optimal cut-off points were determined using Youden's index. Sensitivity, specificity, and AUC were calculated. Statistical analyses were performed using SPSS Statistics version 22 (IBM Corp., Armonk, NY, IMM Corp). All the statistical tests were two-sided with a statistical significance level defined as *p*-value <0.05.

## Results

We recruited 398 participants. Table 1 shows the general characteristics of the participants according to the IR and non-IR groups. Among the 398 subjects, 123 (30.9%) were in the IR group. There were 139 men (35%) and 259 women (65%), and the mean age was 64.43 ± 8.45 years. There were no significant differences between the IR and non-IR groups with respect to age, creatinine level, sex, and smoking status. Significant differences were found in SBP; DBP; BMI; WC; FPG, HDL-C, LDL-C, total cholesterol, TG, and WBC levels; DM; HTN; and hyperlipidemia. The overall percentage of participants reporting current smoking was 10.6%. The average BMI was 24.54 ± 3.57 kg/m^2^; WC, 84.99 ± 9.62 cm; and, WBC count, 6.04 ±1.59 ×1,000/uL. The mean SBP and DBP were 129.57 ± 16.72 and 76.94 ± 11.36 mmHg, respectively. Overall, the mean FPG, HDL-C, LDL-C, total cholesterol, and TG levels were 95.54 ± 22.32, 54.51 ± 13.91, 118.47 ± 32.16, 197.19 ± 35.69 and 121.20 ± 62.93 mg/dL, respectively.

**Table 1:** General characteristics of the study population according to IR group and non-IR group.

**Variables**	**Total**	**Insulin Resistance**		
		**IR group (≥2)**	**Non-IR group (<2)**	**p value**
	**(n** **=** **398)**	**(n** **=** **123)**	**(n** **=** **275)**	
Age (year)	64.43 ± 8.45	63 [59, 71]	63 [58, 70]	0.68
SBP (mmHg)	129.57 ± 16.72	133.97 ± 16.49	127.60 ± 16.47	<0.001
DBP (mmHg)	76.94 ± 11.36	78.91 ± 10.66	76.06 ± 11.57	0.02
BMI (kg/m^2^)	24.54 ± 3.57	26.70 [23.5, 28.4]	23.30 [21.8, 25.6]	<0.001
Waist circumference (cm)	84.99 ± 9.62	90 [83, 96]	82 [77, 88]	<0.001
Creatinine (mg/dL)	0.77 ± 0.42	0.71 [0.58, 0.86]	0.67 [0.57, 0.84]	0.26
FPG (mg/dL)	95.54 ± 22.32	101 [91, 120]	87 [81, 94]	<0.001
HDL-C (mg/dL)	54.51 ± 13.91	47 [40, 56]	55 [47, 66]	<0.001
HOMA-IR index	1.86 ± 1.39	2.84 [2.31, 3.83]	1.17 [0.83, 1.52]	<0.001
LDL-C (mg/dL)	118.47 ± 32.16	112.11 ± 29.83	121.31 ± 32.80	0.01
Total Cholesterol (mg/dL)	197.19 ± 35.69	191.46 ± 34.08	119.75 ± 36.16	0.03
Triglyceride (mg/dL)	121.20 ± 62.93	131 [105, 180]	96 [73, 126]	<0.001
WBC (1000/uL)	6.04 ± 1.59	6.3 [5.7, 7.4]	5.7 [4.8, 6.6]	<0.001
Men, *n* (%)	139 (34.9)	41 (33.3)	98 (35.6)	0.66
Current smoking, *n* (%)	42 (10.6)	12 (9.8)	30 (10.9)	0.73
HTN, *n* (%)	199 (50.0)	84 (68.3)	115 (41.8)	<0.001
DM, *n* (%)	77 (19.3)	48 (39.0)	29 (10.5)	<0.001
Hyperlipidemia, *n* (%)	259 (65.1)	93 (75.6)	166 (60.4)	0.003

*Clinical characteristics are expressed as mean ± SD or median [Q1, Q3] for continuous variables and n (%) for categorical variables. P-value were derived from independent t-test and Mann-Whitney U test for continuous variables and chi-square test for categorical variables.*

*SBP, systolic blood pressure; DBP, diastolic blood pressure; BMI, body mass index; FPG, fasting plasma glucose; HDL-C, high-density lipoprotein cholesterol; LDL-C, low-density lipoprotein cholesterol; WBC, white blood cell count; HTN, hypertension; DM, diabetes mellitus*.

**Figure 1:**
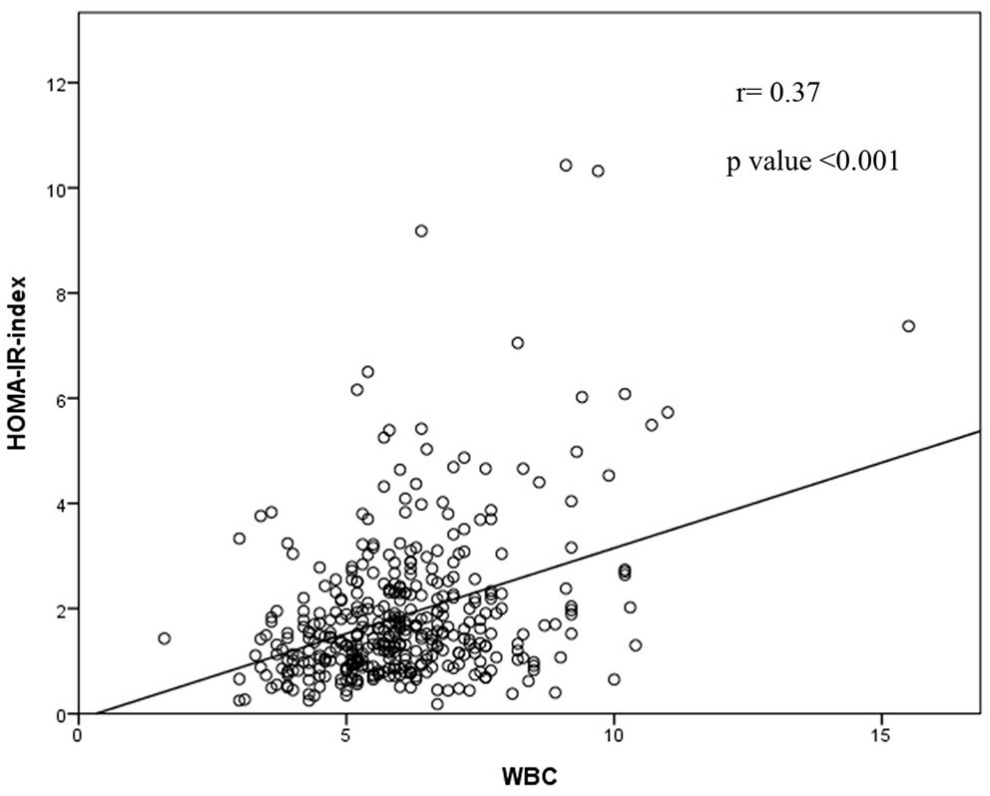
Representation of the correlation analysis: there was a trend toward a positive correlation between HOMA-IR index and WBC.

Table 2 demonstrates the correlations between the HOMA-IR index and the cardiometabolic risk factors. The WBC count remained positively associated with IR before and after adjusting for age. The WBC count showed a stronger correlation with HOMA-IR than most of the risk factors analyzed. Figure 1 demonstrates the association of WBC and HOMA-IR index with a Pearson's correlation coefficient of 0.37.

**Table 2:** The correlation between HOMA-IR index and cardio-metabolic risk factors.

**Variables**	**HOMA-IR index (*****n*** **=** **398)**			
	**Unadjusted**		**Adjusted for age**	
	**Pearson's** **coefficient**	***p*-** **value**	**Pearson's** **coefficient**	***p*-** **value**
Age (year)	0.02	0.70	NA	NA
SBP (mmHg)	0.17	0.001	0.17	0.001
DBP (mmHg)	0.09	0.079	0.10	0.06
BMI (kg/m^2^)	0.43	<0.001	0.433	<0.001
Waist circumference (cm)	0.41	<0.001	0.41	<0.001
FPG (mg/dL)	0.50	<0.001	0.50	<0.001
HDL-C (mg/dL)	−0.31	<0.001	−0.31	<0.001
LDL–C (mg/dL)	−0.10	0.06	−0.09	0.06
TG (mg/dL)	0.33	<0.001	0.33	<0.001
WBC (1000/uL)	0.37	<0.001	0.37	<0.001

*SBP, systolic blood pressure; DBP, diastolic blood pressure; BMI, body mass index; FPG, fasting plasma glucose; HDL-C, high-density lipoprotein cholesterol; LDL-C, low-density lipoprotein cholesterol; TG, triglyceride; WBC, white blood cell count*.

The results of the multivariate logistic regression analyses are given in Table 3, where IR is the dependent variable and WBC count is the independent variable. The odds ratios (ORs) (95% confidence interval [CI]) of IR in terms of WBC count are presented in Table 3. In Model 1, the ORs (95% CI) were calculated after adjusting for sex. In Model 2, the ORs (95% CI) were calculated after adjusting for sex, age, and BMI. In Model 3, we examined the relationship between WBC count and IR after adjusting for additional confounding variables such as HTN, DM, and hyperlipidemia. In Model 4, we adjusted for sex, age, BMI, current smoking status, HTN, DM, and hyperlipidemia. In all the four models, BMI, DM, and WBC count were significantly associated with IR. In Model 4, BMI (adjusted odds ratio [aOR]: 1.25; 95% CI: 1.15–1.35; *p* <0.001), HTN (aOR: 1.87, 95% CI: 1.11–3.17; *p*-value <0.05), DM (aOR: 5.4, 95% CI: 2.93–9.93; *p*-value <0.001), and WBC count (aOR: 1.5, 95% CI: 1.25–1.81; *p*-value <0.001) were all significantly associated with IR.

**Table 3:** Association between WBC levels and insulin resistance.

**Variables**	**Odds ratio**	**95% C.I**.	***p*-value**
**Model 1**			
Sex (men vs. women)	0.68	0.42–1.10	0.12
WBC (1000/uL)	1.54	1.32–1.80	<0.001
**Model 2**			
Sex (men vs. women)	0.58	0.34–0.97	0.04
Age (year)	1.01	0.98–1.04	0.52
BMI (kg/m^2^)	1.27	1.18–1.37	<0.001
WBC (1000/uL)	1.54	1.30–1.82	<0.001
**Model 3**			
Sex (men vs. women)	0.61	0.35–1.06	0.08
Age (year)	0.99	0.96–1.03	0.69
BMI (kg/m^2^)	1.25	1.15–1.35	<0.001
HTN (yes vs. no)	1.87	1.11–3.16	0.02
DM (yes vs. no)	5.34	2.91–9.80	<0.001
Hyperlipidemia (yes vs. no)	1.38	0.79–2.40	0.26
WBC (1000/uL)	1.47	1.23–1.77	<0.001
**Model 4**			
Sex (men vs. women)	0.67	0.38–1.20	0.18
Age (year)	0.99	0.96–1.02	0.59
BMI (kg/m^2^)	1.25	1.15–1.35	<0.001
Smoking (yes vs. no)	0.61	0.24–1.51	0.28
HTN (yes vs. no)	1.87	1.11–3.17	0.02
DM (yes vs. no)	5.40	2.93–9.93	<0.001
Hyperlipidemia (yes vs. no)	1.43	0.82–2.51	0.21
WBC (1000/uL)	1.50	1.25–1.81	<0.001

*BMI, body mass index; HTN, hypertension; DM, diabetes mellitus; WBC, white blood cell count; CI, confidence interval*.

**Figure 2:**
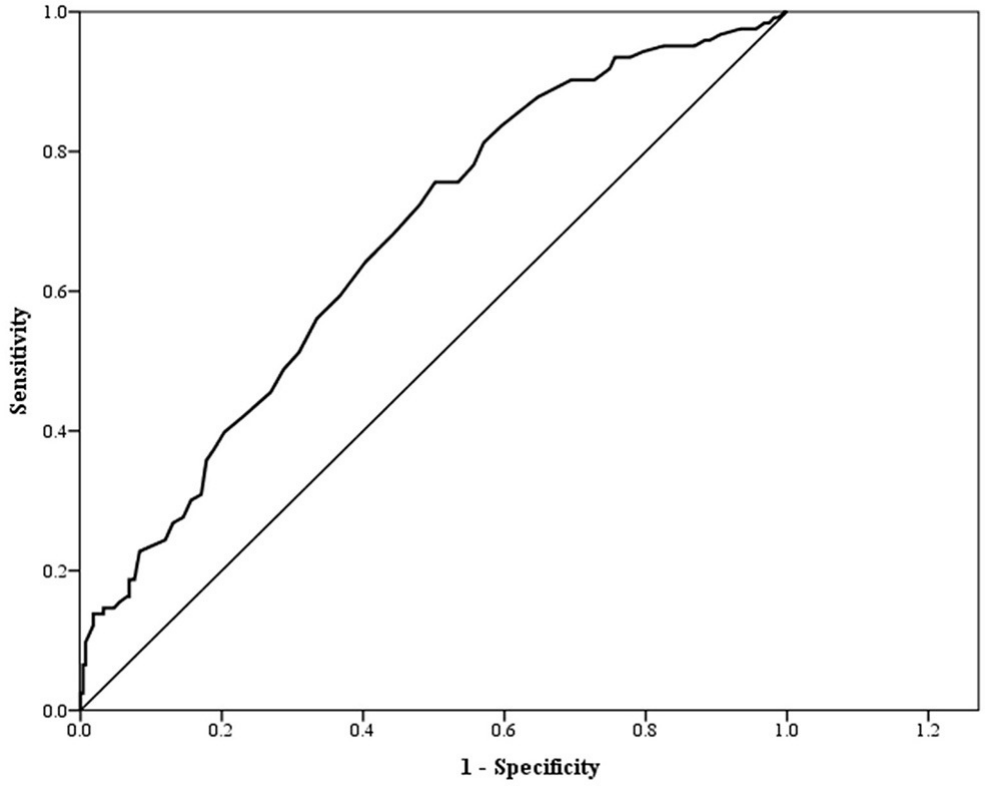
ROC curve for WBC as a predictor of insulin resistance.

The AUC of WBC for predicting IR was 0.67 (Table 4, Figure 2). The optimal cut-off point for WBC for predicting IR was 5.65 (1,000/uL), with a corresponding sensitivity and specificity of 75.6 and 49.8%, respectively.

**Table 4:** The areas under ROC curve (AUC), sensitivity, specificity by the optimized cut-off points for WBC in predicting HOMA-IR index.

**Variables**	**AUC (95% CI)**	***p* value**	**Cut-off point**	**Sensitivity**	**Specificity**
WBC (1,000/uL)	0.67	<0.001	5.65	0.756	0.498

*WBC, white blood cell count; ROC curve, receiver operating characteristic curve; CI, confidence interval*.

## Discussion

In this cross-sectional community-based study, we found that a high WBC count was positively related to IR after adjusting for potential confounding variables in the middle-aged and older populations in Taiwan.

Several studies have reported a positive correlation between WBC count and IR (16, 18, 20, 21), which is consistent with our findings. However, few studies have used the WBC count to identify the risk of IR. Park et al. (22) recently suggested that the WBC count can facilitate the identification of children and adolescents with IR. However, to our knowledge, it has not been used in adults.

Clinical prediction rules have become increasingly common in the improvement of healthcare delivery. This study aimed to use a simple biomarker as a clinical tool for evaluating IR in middle-aged and older populations. Therefore, we used logistic regression to explore the association between IR and the WBC count. Previous studies have shown that WBC count is a risk factor for metabolic syndrome (23), T2DM (24) and cardiovascular disease (25). It has been suggested that the link between WBC count and cardiovascular disease may be represented by a decrease in insulin sensitivity (25, 26). The association between WBC count and IR was significant in the middle-aged and older populations in Taiwan, even after adjusting for multiple covariates.

In the present study, IR was measured by HOMA-IR, an extensively validated tool to quantify IR, and our cut-off value (2.0) was similar to that of a study in 1,156 Caucasians (2.29) (27, 28). The covariates (BMI, WC, FPG level, HDL-C level, TG level, and WBC count) were all significantly associated with IR, regardless of age, through Pearson's correlation coefficient. In our clinical prediction models, the WBC count remained significantly associated with IR after adjusting for covariates such as age, sex, BMI, current smoking status, HTN, DM, and hyperlipidemia. This result reinforces the relationship between IR and WBC count; therefore, we suggest that WBC count could be used to identify IR.

In previous studies, Chao et al. (29) found that the threshold value of WBC count for predicting the future development of metabolic syndrome was 5 ×1,000/uL. Oda et al. (30) reported that the WBC count threshold values for predicting metabolic syndrome were 5 ×1,000/uL for women and 5.63 ×1,000/uL for men. Twig et al. (21) reported that patients with WBC counts above 6.9 ×1,000/uL were at a 52% increased risk of diabetes as compared to those in the lowest quintile. In our study, the WBC count threshold value for identifying IR in middle-aged and older Taiwanese was 5.65 ×1,000/uL.

In our study, the AUC value for identifying IR (as measured by HOMA-IR) with WBC count was 0.67, and the optimal threshold was 5.65 ×1,000/uL, with a corresponding sensitivity and specificity of 75.6% and 49.8%, respectively. We considered WBC count to be a potential marker for IR assessment. We suggest that WBC count could be an alternative marker used in combination with HOMA-IR to identify IR individuals in this population.

Even though we obtained an AUC of 0.67, which showed sufficient accuracy of the marker (31),we would still recommend using the WBC count alone for IR assessment with caution, as the WBC count may be influenced by an array of factors including acute infection, bone marrow disorders, and certain medications (32).

Our study had several strengths. First, the features of this study include a clear design, a sufficient sample size, a comprehensive inclusion of relevant confounders, and a well-performed data analysis. Second, the novelty of this study was, from a community approach, to report different cut-off values of WBC count among the middle-aged and older populations to better predict insulin resistance in these two specific age groups. Third, no similar studies have search investigated this topic in a community-dwelling population in Taiwan. There are several limitations to our study. First, there was lack of information on alcohol intake, regular physical exercise, and family history of diabetes in the study population. Second, the cross-sectional design used in this study made it difficult to explore the causal relationship between WBC count and IR in the study population. Third, all the participants were volunteers from a community in northern Taiwan, which may have led to a possible selection bias.

In conclusion, elevated WBC was significantly associated with IR in the middle-aged and older populations in Taiwan. However, WBC count may not only serve as a biomarker for early identification of IR but may also be used as a surrogate marker for excluding IR. Future research is warranted to further investigate the use of WBCs for predicting IR in a larger population.

## Data Availability Statement

The raw data supporting the conclusions of this article will be made available by the authors, without undue reservation.

## Ethics Statement

The studies involving human participants were reviewed and approved by Chang Gung Medical Foundation Institutional Review Board (102-2304B). The patients/participants provided their written informed consent to participate in this study.

## Author Contributions

J-YC contributed to the conception and design of the research. J-YC and M-TT were involved in data acquisition, analysis and interpretation, drafted, and revised the manuscript critically for important intellectual content. Y-HC and Y-CL were involved in data cleaning and follow-up. All authors have read and agreed to the published version of the manuscript.

## Funding

This work was supported by Chang Gung Memorial Hospital, Grant Numbers CORPG3C0171, CORPG3C0172, and CZRPG3C0053.

## Conflict of Interest

The authors declare that the research was conducted in the absence of any commercial or financial relationships that could be construed as a potential conflict of interest.

## Publisher's Note

All claims expressed in this article are solely those of the authors and do not necessarily represent those of their affiliated organizations, or those of the publisher, the editors and the reviewers. Any product that may be evaluated in this article, or claim that may be made by its manufacturer, is not guaranteed or endorsed by the publisher.

## Abbreviations

HOMA-IR: Homeostasis model assessment of insulin resistance
IR: Insulin resistance
T2DM: Type 2 Diabetes mellitus
WBC: White blood cell count
CRP: C-reactive protein
BMI: Body Mass Index
WC: Waist Circumference
BP: Blood pressures
FPG: Fasting plasma glucose
HDL-C: High-density lipoprotein cholesterol
LDL-C: Low-density lipoprotein cholesterol
TG: Triglyceride
ROC: Receiver operating characteristic
AUC: Area under the ROC curve
HTN: Hypertension
aOR: Adjusted odds ratio
IDF: International Diabetes Federation.
